# A new species of *Metacyclops* Kiefer, 1927 (Copepoda, Cyclopidae, Cyclopinae) from the Chihuahuan desert, northern Mexico

**DOI:** 10.3897/zookeys.287.4358

**Published:** 2013-04-11

**Authors:** Nancy F. Mercado-Salas, Eduardo Suárez-Morales, Alejandro M. Maeda-Martínez, Marcelo Silva-Briano

**Affiliations:** 1 El Colegio de la Frontera Sur (ECOSUR) Unidad Chetumal, A. P. 424. Chetumal, Quintana Roo 77014, Mexico; 2 Centro de Investigaciones Biológicas del Noreste, S. C., Instituto Politécnico Nacional 195, Playa Palo de Santa Rita Sur, La Paz, Baja California Sur, 23060, Mexico; 3 Universidad Autónoma de Aguascalientes, Aguascalientes 20100, México

**Keywords:** Arid environments, Cyclopoida, freshwater zooplankton, inland water crustaceans, copepod taxonomy

## Abstract

A new species of the freshwater cyclopoid copepod genus *Metacyclops* Kiefer, 1927 is described from a single pond in northern Mexico, within the binational area known as the Chihuahuan Desert. This species belongs to a group of *Metacyclops* species with a 3443 spine formula of swimming legs. It is morphologically similar to *Metacyclops lusitanus* Lindberg, 1961 but differs from this and other congeners by having a unique combination of characters, including a caudal rami length/width proportion of 3.5–3.8, a innermost terminal seta slightly longer than the outermost terminal seta, intercoxal sclerites of legs 1-4 naked, a strong apical spine of the second endopodal segment of leg 1 and one row of 6-8 small spinules at the insertion of this spine. The finding of this species represents also the first record of the genus in Mexico and the third in North America, where only two other species, *Metacyclops gracilis* (Lilljeborg, 1853)and *Metacyclops cushae* Reid, 1991 have been hitherto reported. This is also the first continental record of a species of *Metacyclops* from an arid environment in the Americas. This species appears to be endemic to the Chihuahuan Desert, thus emphasizing the high endemicity of this area.

## Introduction

The freshwater copepod genus *Metacyclops* Kiefer, 1927 was revised by Herbst (1988), who recognized 47 species. Many species have been added from investigations in different regions of the world (Brancelj 1987, Reid 1987, 1991, Herbst 1990, Rocha 1994, Galassi and Pesce 1994, Pesce et al. 1996, Karanovic 2004a) and the genus is currently known to contain more than 62 nominal species and subspecies (Dussart and Defaye 2006). In the Americas, the genus is represented by 24 species, most of them occurring in Central and South America; it appears to be quite less diverse in North America, where only two species have been recorded: *Metacyclops gracilis* (Lilljeborg, 1853) from Minnesota (Herrick 1895), Maryland (Wilson 1932), and a cave in Texas (Davis 1979),and *Metacyclops cushae* Reid, 1991 (Reid 1991) from a temporary pool in New Orleans Parish, Louisiana. It is probable that the diversity of the genus in North America could be underestimated as result of inadequate sampling of suitable non-planktonic habitats and because of the exclusion of the genus from regional keys (Reid 1991, Dussart and Defaye 2006, Gaviria and Aranguren 2007, J. Reid pers. comm.).

Lindberg (1961) separated the genus into two distinct groups: 1) the “*minutus-planus*” group with one spine on the third endopodal segment of the fourth leg and 2) the “*gracilis-mendocinus*” group with two such spines. Recent taxonomic works (Herbst 1988, Reid 1987, 1991, Fiers 2001, Karanovic 2004a, 2004b) recognize four groups based on the spine formula of the terminal exopodite segment of legs 1-4 (designated as spine formula of legs 1-4). The first group, with a 3443 spine formula contains 52 of the 62 species of the genus. The second (3442) and third (3433) groups each contain one species, *Metacyclops mortoni* Pesce, De Laurentiis and Humphreys, 1996 and *Metacyclops cushae* Reid, 1991, respectively. The fourth group among the species of *Metacyclops* is the *trispinosus-*group, with a 3333 spine formula (Karanovic 2004a, 2004b). Karanovic (2004b) stated that the *trispinosus-*group is an easily recognizable group of species with an Eastern Gondwana connection (Africa, India, Australia, and New Zealand). Until recently, it contained 8 species but Karanovic et al. (2011) reallocated six of them in the new genus *Pescecyclops* (*Pescecyclops pilbaricus* [Karanovic, 2004],*Pescecyclops laurentiisae* [Karanovic, 2004],*Pescecyclops pilanus* [Karanovic, 2004],*Pescecyclops arnaudi* [G. O. Sars, 1908],*Pescecyclops monacanthus* [Kiefer, 1928]and *Pescecyclops kimberlyi* [Karanovic, 2004] ), distinguished by the presence of three spines on the distal exopodal segment of all swimming legs, only one apical spine on the fourth leg endopod, and the absence of sexual dimorphism. Thus, *Metacyclops trispinosus* Dumont, 1981 and *Metacyclops margaretae* (Lindberg, 1938) are the only two species of the *trispinosus*-group that were retained in the genus *Metacyclops*.

Karanovic (2004a, 2004b) stated that many morphological features in *Metacyclops* have an extreme range of variation, which raises the question as to its monophyly and taxonomic validity. It is highly possible that a more detailed examination of the fifth leg, along with many other currently neglected characters will result in splitting the genus *Metacyclops* into several different genera. The recent work by Karanovic et al. (2011) included a cladistic analysis and a taxonomic revision of the Australian species. These authors formally recognized the “*Metacyclops* complex” and provided evidence to state that *Metacyclops* s. str. is polyphyletic in nature and that some species (like *Metacyclops cushae*) are closer to other genus than they are to *Metacyclops*.

Most of the surveys on the Mexican cyclopoid copepod fauna have dealt with the central and southern regions (Suárez-Morales and Reid 1998, Suárez-Morales et al. 2002). Recent research efforts in arid and semi-arid regions of Central and Northern Mexico (Dodson and Silva-Briano 1996, Mercado-Salas et al. 2006, 2009, Suárez-Morales and Walsh 2009; Mercado-Salas and Suárez-Morales 2009, 2011) indicate that the copepod fauna of these habitats is more diverse than previously thought and that it deserves further study. During a recent revision of zooplankton collections from arid areas of Northern Mexico, several specimens of cyclopoid copepods were identified as representing an undescribed species of *Metacyclops*. The significance of this finding is discussed in terms of currently known diversity and distributional patterns of the genus in the Americas.

## Methods

During the development of a project to estimate the diversity of cyclopoid copepods in arid and semi-arid regions of Mexico, zooplankton samples were collected between 1981 and 2009 in more than 500 water bodies from six states of Central and Northern Mexico (Aguascalientes, Chihuahua, Coahuila, Durango, San Luis Potosí, and Zacatecas). Samples were collected using a conical standard plankton net (250 mm diameter and 50 µm-mesh size) hauled near the shoreline of water bodies. The biological material was then fixed and preserved in 4% formalin solution. Copepods were sorted out from the original samples and then transferred to 70% ethanol with a drop of glycerine for long term preservation. Several female and male specimens of a cyclopoid copepod were collected from Coahuila in northern Mexico. These copepods were tentatively identified as *Apocyclops panamensis* (March, 1913). A second, closer examination of these specimens was performed in the laboratory and differences with respect to *Apocyclops panamensis* motivated a deeper analysis which revealed these specimens as members of *Metacyclops*. The taxonomically relevant characters of this genus were evaluated following Reid (1987, 1991), Herbst (1988), Rocha (1994), Karanovic (2004a, 2004b), and Karanovic et al. (2011). Specimens were processed for taxonomical examination following Reid and Williamson (2010). Three females were dissected and 10 females were prepared for SEM examination with a JEOL LV 5900 microscope at facilities of the Universidad Autónoma de Aguascalientes, Mexico. The SEM processing included dehydration in progressively higher ethanol concentrations (60, 70, 80, 96, 100%), drying, and gold coating following standard methods. All dissected specimens were mounted in semi-permanent slides with glycerine sealed with Entellan®, a commercial, fast drying mounting medium and sealant. Scaled illustrations were done at 100X magnifications with a drawing tube mounted on a standard Olympus CX31 microscope. Type specimens were deposited in the collection of zooplankton held at El Colegio de la Frontera Sur, in Chetumal, Mexico (ECO-CH-Z) and in the Laboratorio de Ecología of the Universidad Autónoma de Aguascalientes, Mexico.

## Results

### Order Cyclopoida Rafinesque, 1815 Family Cyclopidae Rafinesque, 1815 Subfamily Cyclopinae Rafinesque, 1815 Genus *Metacyclops* Kiefer, 1927

#### Metacyclops deserticus

Mercado-Salas & Suárez-Morales sp. n.

urn:lsid:zoobank.org:act:440E0C58-17CB-4A7F-A841-DC46403F02AB

http://species-id.net/wiki/Metacyclops_deserticus

Figures 1 5

##### Material examined.

Holotype. Adult ♀, specimen dissected, mounted in glycerin sealed with Entellan (ECO-CH-Z-08585). Allotype. Adult ♂, dissected and mounted in glycerin and sealed with Entellan (ECO-CH-Z-08586). Paratypes.15 adult ♀♀ specimens, undissected, ethanol-preserved, vial (ECO-CH-Z-08587). Original plankton samples containing several additional specimens, and the SEM-processed specimens are deposited in the collection of M. Silva-Briano, Laboratorio de Ecología of the Universidad Autónoma de Aguascalientes, Mexico. Samples from the type locality were collected by Alejandro Maeda-Martínez in October 10, 1981.

##### Type locality.

Ephemeral pond at El Refugio bridge, Cerro Bola, Km 70, east of Torreón city, federal highway 40, Coahuila (25°35'02"N, 102°45'02"W).This pond is located in a desertic plain in the southwest margin of the ancient Laguna de Mayrán, a system which is part of the endorheic drainage of the Nazas and Parras rivers. The altitude of the type locality is about 1,100 meters above sea level and the average annual precipitation in the area is 200 mm. At the moment of sampling the surface area of the pond was about 55 meters long and 25 meters wide, the water had an average depth of 50 cm and 80 cm at its deepest point.

##### Etymology.

The specific epithet makes reference to the arid habitat from which this species was collected. It was used to emphasize that it is the first American record from arid conditions.

##### Descriptions.

**Figure 1.**
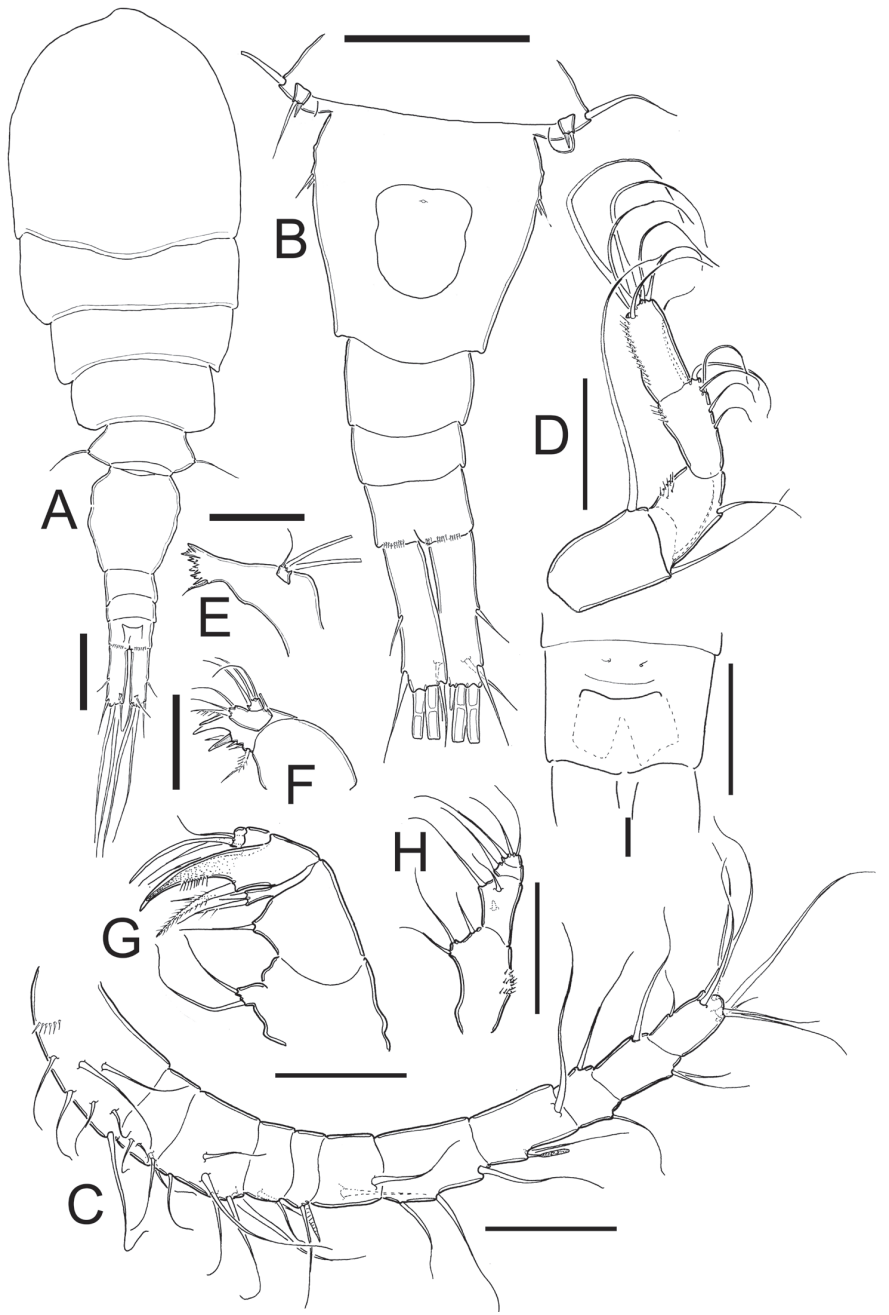
*Metacyclops deserticus* sp. n., female holotype from Coahuila, Mexico. **A** habitus, dorsal view **B** urosome, ventral view **C** antennule **D** antenna **E** mandible **F** maxillule **G** maxilla **H** maxilliped **I** anal operculum. Scales bars **A–B**= 100µm; **C–I**= 50 µm.

*Female*: Habitus as in Fig. 1A (dorsal view) and Fig. 4A (lateral view).Length of holotype 0.87 mm from anterior end of cephalothorax to posterior margin of caudal rami (range=0.72–0.87 mm; mean=0.80 mm; n=9). Body robust, cephalothorax relatively long, slightly expanded laterally at midlength of cephalosome in dorsal view; lateral margins of pedigers 3 and 4 straight, produced posteriorly. Cephalothorax length= 0.55 mm, representing 63% of total body length. Dorsal surface smooth, antennules not reaching distal margin of first pediger. Urosome (excluding caudal ramus) (Fig. 1B) representing 37% of body. Posterior margins of genital double-somite, free urosomites, and anal somite smooth both dorsally and ventrally. Relative length of each urosomite (proximal to distal) as: 65.4: 10.3: 10.3: 14.1=100. Genital double-somite (Fig. 5E) representing 17% of body length (excluding caudal rami), somite about 1.1 times longer than broad, with maximum width at proximal half; ventral and dorsal surfaces smooth. Anterior half of genital double-somite expanded laterally. Seminal receptacle with a reduced and narrow anterior part, posterior part rounded and expanding along the somite. Anal somite with distal rows of spines at insertion points of each caudal rami on ventral and dorsal margins. Anal operculum (Fig. 1I) slightly rounded and smooth.

*Caudal ramus* (Fig. 1B, 5F): Ramus representing 8.2% of total body length and 0.3 times as long as urosome. Length/width ratio= 3.5–3.8. Inner and outer margins smooth. Lateral caudal seta (II) inserted at 53% of total length of caudal rami. Outermost terminal seta (III) without ornamentation at point of insertion and 0.6–0.7 times as long as caudal ramus. Dorsal seta (VII) relatively short, 0.4–0.5 times as long as caudal ramus. Innermost terminal seta (VI) about 0.5 times as long as caudal ramus. Innermost terminal seta (VI) about 0.8–0.84 times outermost terminal seta (III). All terminal caudal setae plumose.

*Antennule* (Fig. 1C, 4A): 11-segmented in all specimens examined, armature per segment as follows (s=seta, sp= spine, ae=aesthetasc): 1(7s), 2(4s), 3(6s), 4(2s), 5(1s +1sp), 6(2s), 7(3s), 8(2s + 1ae), 9(2s), 10(3s), 11(7s). Antennule not reaching posterior margin of first thoracic somite.

*Antenna* (Fig. 1D, 4C): Four-segmented, basis without cuticular ornamentation, armed with long exopodal seta and two basipodal setae of different size, outer seta 1.6 times longer than inner seta. First endopodal segment with single outer seta and inner group of spinules. Second segment with 6 setae; inner margin with longitudinal row of spinules. Third endopodal segment with 6 terminal setae; inner margin with row of spinules.

*Mandible* (Fig. 1E): Gnathobase with 7 strongly chitinized teeth and dorsal seta armed with inner row of spinules. Palp reduced, with 2 long and 1 short setae, the later not reaching half-length of former two.

*Maxillule* (Fig. 1F): Precoxal arthrite with 3 strong chitinized claws and 2 spiniform setae on frontal side. Palp 2-segmented, proximal segment armed with 3 inner setae and outer exopodal seta. Distal segment of palp armed with 3 setae.

*Maxilla* (Fig. 1G): Precoxa and coxa not fused; precoxal endite armed with two strong biserially setulated setae. Coxal surface naked, proximal endite well developed, with two subequal apical setae. Claw-like distal endite well developed, with row of 6 spinules and basal seta. Endopodite 2-segmented, proximal segment with 2 robust setae, distal segment with single seta.

*Maxilliped* (Figs. 1H, 4D): Four-segmented. Syncoxa with 3 spiniform setae along inner margin: proximal one without ornamentation at insertion, middle one longest, more than twice as long as the other setae. Basis with 2 spiniform setae and transverse row of spines. Endopod reduced, 2-segmented, first segment with single lightly spinulate seta. Second endopodal segment armed with spiniform proximal seta and 2 slender setae.

*Legs P1-P4*: with naked intercoxal sclerites, distal margins with rounded projections. All endopodal and exopodal setae slender and plumose. Armature formula of all swimming legs as in Table 1.

**Figure 2.**
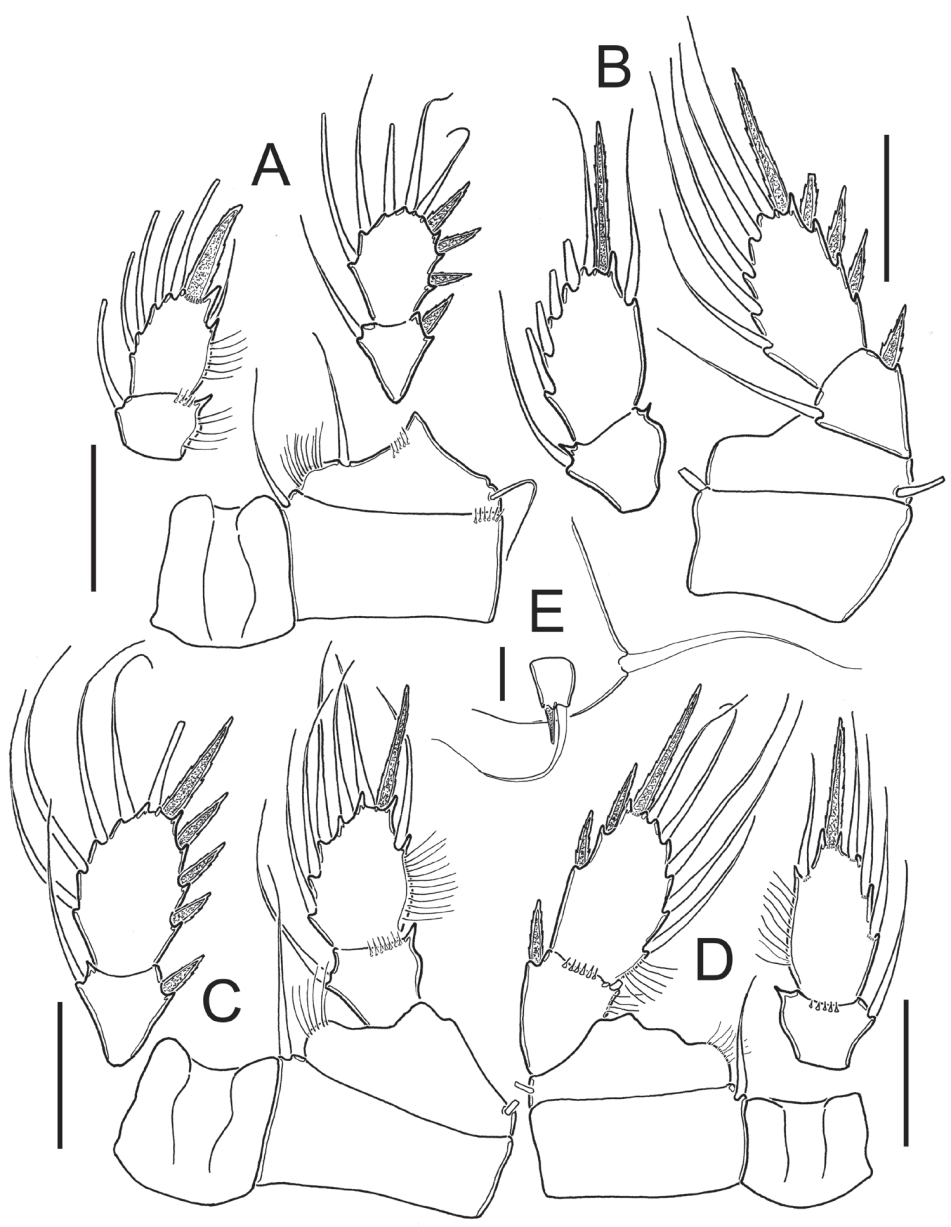
*Metacyclops deserticus* sp. n., female holotype from Coahuila, Mexico. **A** Leg 1 **B** Leg 2 **C** Leg 3 **D** Leg 4 **E** Leg 5. Scales bars **A–D**= 50 µm; **E**= 10 µm.

*Leg 1*(Fig. 2A, 5A): Coxa with inner seta and transverse row of 6 spinules on distal outer margin. Basis with inner row of short setae and long slender basipodal seta, reaching middle margin of second endopodal segment, row of hair-like setules along inner margin, row of 5 spines adjacent to insertion of endopodal ramus. Endopod slightly shorter than exopodite. Apical spine of second endopodal segment strong, slightly longer than segment, with spinules at insertion point.

*Leg 2* (Fig. 2B): Coxa with inner seta . Basis with short slender seta on outer margin. Surface of coxa and basis smooth. Endopod slightly shorter than exopodite.

*Leg 3* (Fig. 2C): Coxa with inner seta. Basis with outer seta. Surface of coxa and basis naked. Exopodite slightly longer than endopod.

*Leg 4* (Figs. 2D, 5B): Coxa and basis as in legs 2-3. Endopod shorter than exopodite. Second endopod about two times longer than wide (1.9), with apical spine shorter than bearing segment(0.8 times as long as segment). Spinules at insertion of all elements of second endopodal segment. Second exopodal segment with 2 outer spines and 1 apical spine with small spinules at insertion point.

*Leg 5* (Figs. 1B, 2E, 5C): Basal segment completely fused to somite, dorsal seta stout and plumose, about 1.4 times longer than outer seta of free segment. Free segment subrectangular, 1.2 times longer than wide, inner spine slightly shorter than bearing segment. Outer seta about 4 times longer than inner spine. Inner spine strong and smooth; outer seta plumose on distal half.

*Leg 6* (Figs 1B, 5D): Represented by small, low plate near lateral margin of genital double somite. Leg armed with relatively long plumose seta, and with 2 short, subequal smooth spines.

**Figure 3.**
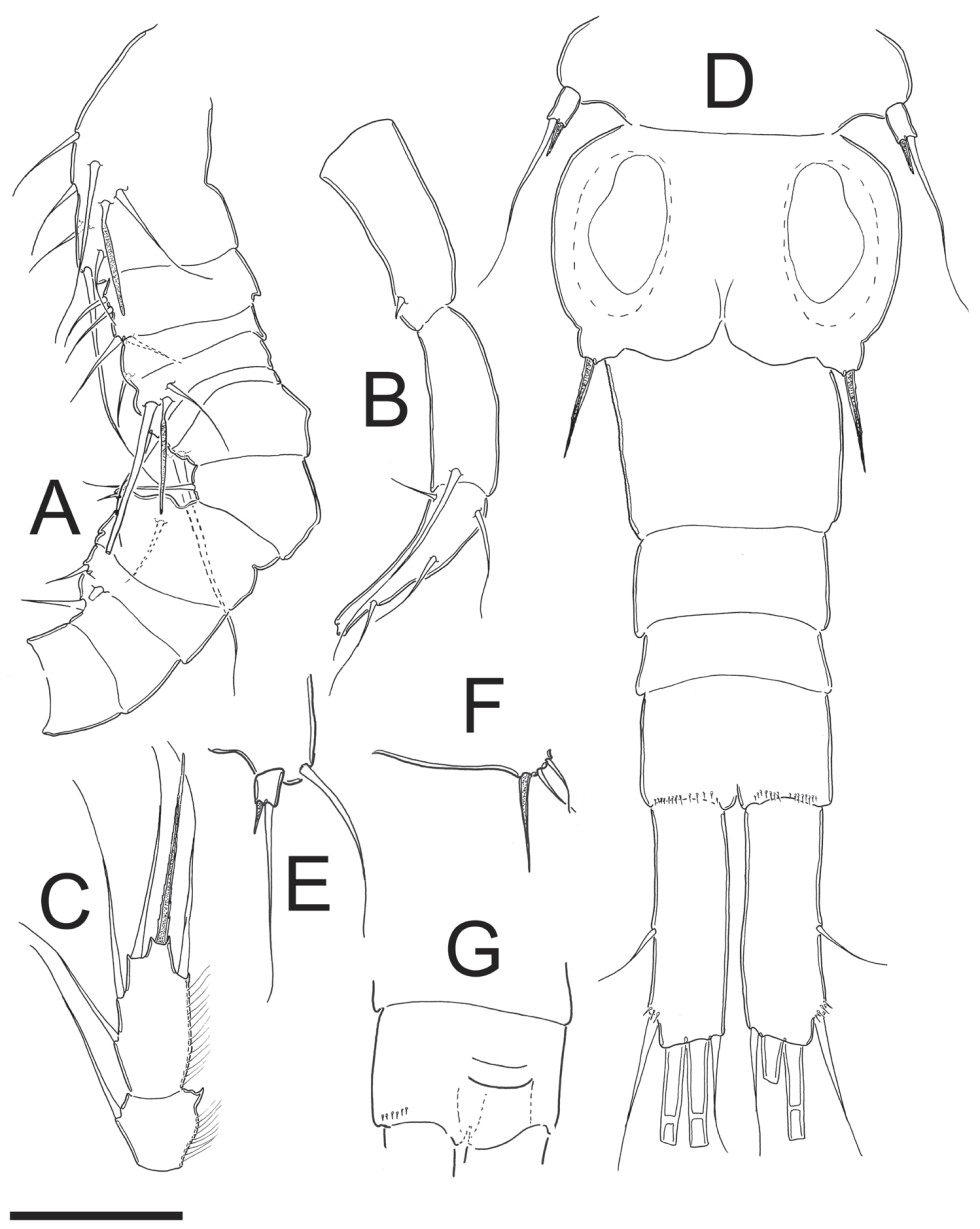
*Metacyclops deserticus* sp. n., male allotype from Coahuila, Mexico. **A** antennule (segments 1–11) **B** antennule (segments 12–14) **C** Endopod P4 **D** urosome **E** Leg 5 **F** Leg 6 **G** Anal operculum. Scales bars **A–G** = 50 µm.

*Male*:Length of allotype 0.58 mm (excluding caudal ramus) (range=0.58–0.64 mm; mean= 0.61mm; n =2). Body slender than in female, cephalothorax relatively long, slightly expanded laterally at midlength of cephalosome in dorsal view; lateral margins of pedigers 3 and 4 straight, produced posteriorly. Cephalothorax length= 0.40 mm, representing 68% of total body length, dorsal surface smooth. Posterior margins of genital somite, free urosomites, and anal somite smooth ventrally (Fig. 3D) and dorsally. Ventral surface of anal somite smooth; distal ventral margin with rows of 13–15 spines at insertion point of caudal rami. Anal operculum (Fig. 3G) slightly rounded, smooth.

*Caudal ramus* (Fig. 3D): Length of ramus 0.07 mm. Length/width ratio= 3.1–3.2. Inner and outer margins smooth, unornamented. Lateral apical seta (II) inserted al 52.3% of total length of caudal ramus. Outermost terminal (III) seta with small spinules at insertion and 0.7–0.8 times as long as caudal ramus. Dorsal seta (VII) longer than in females; about 0.7 times as long as caudal ramus. Innermost terminal seta (VI) 0.5 times as long as caudal ramus and. Innermost terminal seta (III) slightly shorter than outermost terminal seta (VI), III/VI ratio 0.78–0.9. All terminal caudal setae plumose.

*Antennule* (Fig. 3A–B): 14-segmented, geniculate, armature of segments 12, 13 and 14 not seen clearly (they could have more setae). Armature per segment as follows (s=seta; sp= spine ae= aesthetasc): 1(7s+1ae), 2(4s), 3(1s), 4(2s+1ae), 5(1s), 6(1s), 7(1s), 8(1s+ 1sp), 9(2s), 10(1sp), 11(0), 12(1sp), 13(1s), 14(4s).

Antenna, mouthparts and legs 1–3 as in female.

*Leg 4* (Fig. 3C): as in female except for relatively longer exopodite.

*Leg 5* (Fig. 3E): Basal segment completely fused to somite, dorsal seta stout, as long as outer seta of free segment. Free segment subrectangular, 1.5 times longer than wide, spine as long as segment, outer seta about 5 times longer than inner spine. Inner spine strong, smooth; outer seta plumose on distal half.

*Leg 6* (Fig. 3F): Represented by small, low plate near lateral margin of genital somite with relatively strong and long inner spine, two outer setae about the half of length of inner spine. Spine and setae smooth.

**Figure 4.**
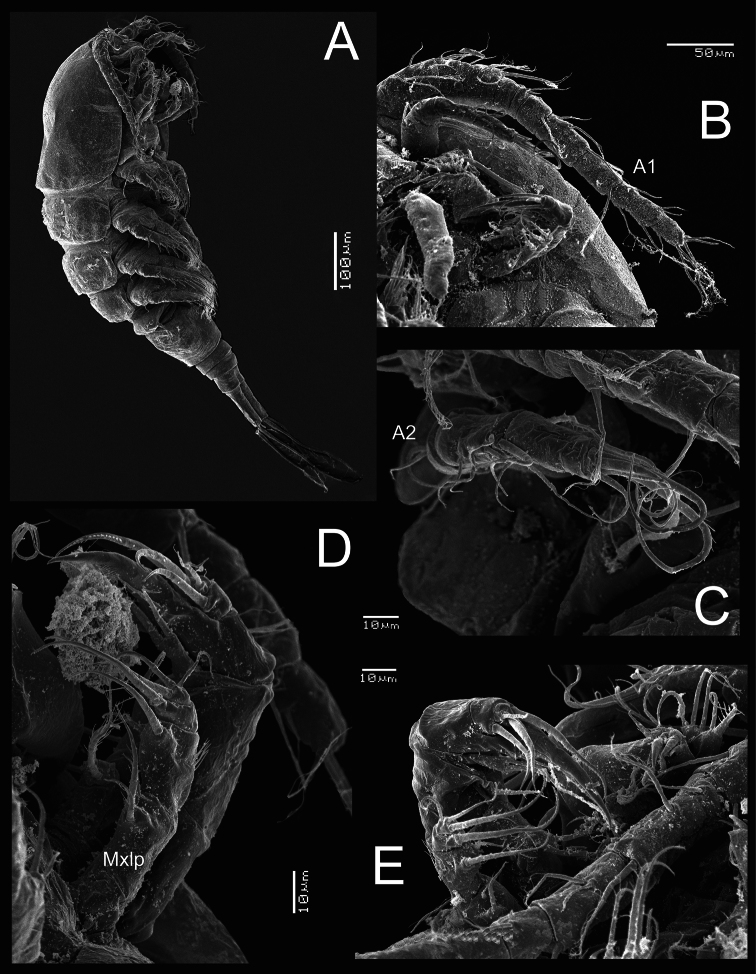
*Metacyclops deserticus* sp. n., SEM-processed female from Coahuila, Mexico. **A** habitus, lateral view **B** antennule **C** antenna **D** maxilliped (lateral view) **E** mouthparts.

**Figure 5.**
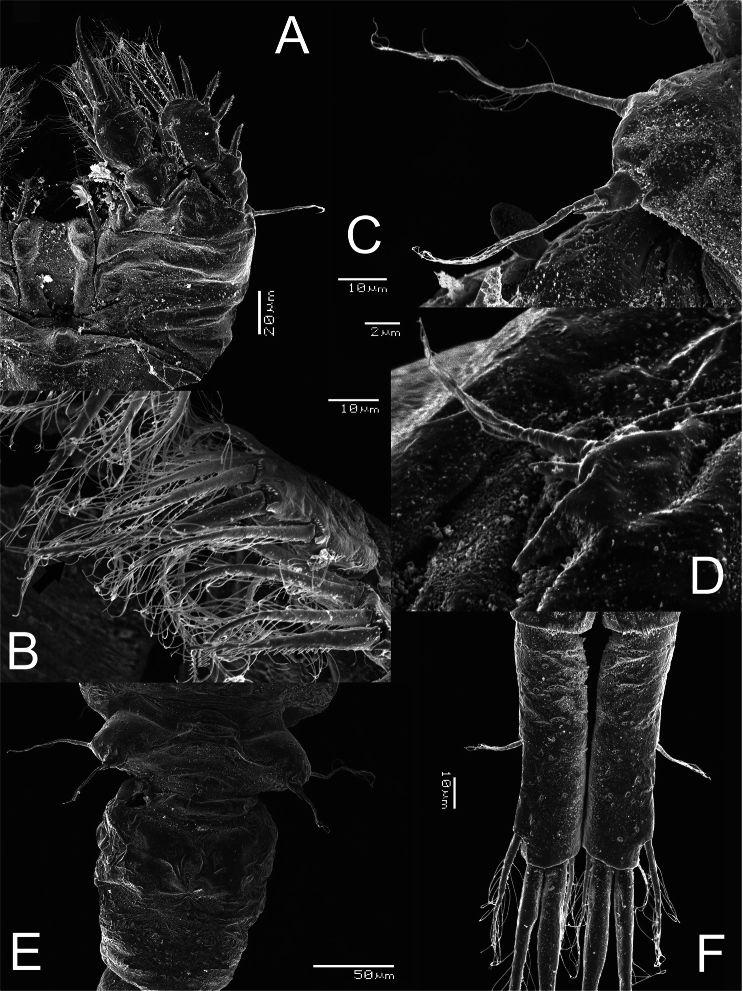
*Metacyclops deserticus* sp. n., SEM-processed female from Coahuila, México. **A** leg 1 **B** endopodite 2 leg 4 **C** leg 5 **D** leg 6 **E** genital double somite, ventral view **F** caudal ramus, ventral.

**Table 1.** Armature of swimming legs 1–4 (spines in Roman numerals, setae in Arabic) of *Metacyclops deserticus* sp. n. Sequence follows external to internal positions.<br/>

	**coxa**	**basis**	**endopodite**	**exopodite**
leg 1	0-1	1-1	0-1;1-I-4	I-1;III-5
leg 2	0-1	1-0	0-1;1-I-5	I-1;IV-5
leg 3	0-1	1-0	0-1;1-I-5	I-1;IV-5
leg 4	0-1	1-0	0-1;1-I-3	I-1; III-5

##### Remarks.

The only two other species of the genus known to occur in North America, *Metacyclops cushae* Reid, 1991 and *Metacyclops gracilis* (Lilljeborg, 1853), are easily distinguishable from the new species. The former species belongs to the “Group C” (Karanovic 2004a, 2004b), with a 3433 spinal formula, being the only species in the group. *Metacyclops gracilis* belongs, like the new species, to Karanovic´s (2004a, 2004b) “Group A”. The new species differs from *Metacyclops gracilis* mainly by its having of one apical spine on the second endopodal segment of leg 4, instead of two such spines present in *Metacyclops gracilis*.

Following the comprehensive key to the known species of *Metacyclops* (Herbst 1988), the new species was tentatively identified as *Metacyclops lusitanus* Lindberg, 1961 from Portugal because both share several characters including: 1) 11-segmented female antennules, 2) margins of all somites smooth, 3) one spine on the apical margin of the second endopodal segment of leg 4, 4) inner apical seta of caudal ramus shorter than the outer seta, 5) length/width proportion of caudal ramus (about. 4.0), 6) apical seta of fifth leg 4 times longer than the apical spine, and 7) apical spine of the second endopodal segment of leg 4 shorter than the segment. However, a closer examination showed several differences between these two species. In *Metacyclops lusitanus* the dorsal caudal seta (VII)/outermost terminal seta (III) length ratio (0.7) differs from that found in the new species (0.8). In addition, the length ratio of the outermost terminal seta(III)/innermost terminal seta (VI) of the caudal ramus differs between these species, in *Metacyclops lusitanus* it is 1.7 vs. 1.1 in the Mexican species. The length ratio of the basipodal seta/total length of endopod of leg 1 slightly differs in these species, this ratio being 0.9 in the *Metacyclops lusitanus* and 0.85 in the new species. Also, the apical spine of the second endopodal segment of leg 1 is clearly stronger in *Metacyclops deserticus* sp. n. than it is in *Metacyclops lusitanus* (see Lindberg 1961, fig. 1b). *Metacyclops deserticus* sp. n. has 6-8 small spinules at the insertion of the apical spine whereas such ornamentation is absent in *Metacyclops lusitanus*.

We also followed Reid’s (1987) key to the American species. Our specimens key down to *Metacyclops curtispinosus* Dussart, 1984. The new species shares several characters with *Metacyclops curtispinosus*, including 11-segmented antennules, also present in *Metacyclops agnitus* Herbst, 1988, *Metacyclops pectiniatus* Shen and Tai, 1964, *Metacyclops subdolus* Pesce, 1978, *Metacyclops hannensis* Defaye, 1992, and *Metacyclops gasparoi* Stoch, 1987. All of them belong to Karanovic´s (2004a, 2004b) “Group A”. The new species shares with *Metacyclops curtispinosus*, *Metacyclops agnitus*, *Metacyclops pectiniatus*, *Metacyclops subdolus*, and *Metacyclops hannensis* the presence of an exopodal seta on antennal basis, clearly differing from *Metacyclops gasparoi* -which lacks the exopodal seta. The naked antennal basis of *Metacyclops deserticus* sp. n. is shared by *Metacyclops curtispinosus*, *Metacyclops agnitus*, *Metacyclops pectiniatus*, and *Metacyclops subdolus* but *Metacyclops hannensis* bears a proximal row of spinules on the inner margin of the antennal basis.

Additional differences of the new species with respect to the American congeners include the length of the apical spine of the second endopodal segment of leg 1/length of segment ratio (1.2), vs. about 0.7 in *Metacyclops curtispinosus* and *Metacyclops agnitus* andabout 0.9 in *Metacyclops subdolus*, *Metacyclops hannensis*,and *Metacyclops gasparoi*. In *Metacyclops deserticus* sp. n., *Metacyclops curtispinosus*, *Metacyclops subdolus*,and *Metacyclops gasparoi*, the length of the basipodal seta of leg 1 exceeds the medial margin of the second endopodal segment of leg, whereas in *Metacyclops hannensis* it exceeds the total length of the endopodite and in *Metacyclops agnitus* it is absent. All these species have naked coxal sclerites of legs 1–4.

The new species shares a similar length/width ratio of the second endopodal segment of leg4 with *Metacyclops curtispinosus*, *Metacyclops agnitus*, *Metacyclops pectiniatus*,and *Metacyclops subdolus* (range= 1.9–2.1), thus differing from the range reported for *Metacyclops hannensis* (1.6–1.7), and *Metacyclops gasparoi* (3.3). There are additional differences in the length ratio of the apical spine of leg 4 second endopodal segment/length of segment; *Metacyclops deserticus* sp. n. shares with *Metacyclops curtispinosus* (a value of about 0.7) whereas this value is different in *Metacyclops pectiniatus*, *Metacyclops subdolus* and *Metacyclops hannensis* (0.9), *Metacyclops agnitus* (1.1) and *Metacyclops gasparoi* (1.4). The length ratio of external seta of leg 4 second endopodal segment/length of apical spine, is about 0.8 in *Metacyclops deserticus* sp. n. and *Metacyclops curtispinosus*, thus differing from *Metacyclops gasparoi* and *Metacyclops hannensis* (0.9–1.1), *Metacyclops subdolus*, *Metacyclops pectiniatus* and *Metacyclops agnitus* (1.2–1.3). An additional difference between these species is the shape of the inner margin of the leg 4 basis. In the new species but also in *Metacyclops curtispinosus*, *Metacyclops agnitus*, *Metacyclops pectiniatus*, and *Metacyclops hannensis* it is rounded *vs*. triangular- in *Metacyclops subdolus* and *Metacyclops gasparoi*.

In addition, the new species differs from its congeners in the length of the external seta of free segment of P5/ inner spine length ratio; in the new species, this ratio is 4.0, whereas it ranges between 2.9 and 3.1 in *Metacyclops subdolus* and between 5.0 and 5.7 in *Metacyclops hannensis*, *Metacyclops pectiniatus* and *Metacyclops agnitus*. In *Metacyclops gasparoi* this valueis 6.6 and in *Metacyclops curtispinosus* it is about 8.0. The proportion between inner spine/length of segment of P5 is a character that also differs among these species. In *Metacyclops curtispinosus* the ratio is 0.3, in the new species it is about 0.8, in *Metacyclops hannensis* and *Metacyclops agnitus* the spine is as long as the segment, in *Metacyclops pectiniatus* and *Metacyclops gasparoi* it is about 1.3, in *Metacyclops subdolus* 1.5 times. Also the length proportion of the seta of fifth leg fused to the segment/outer seta of free segment represents a character that differs between species, in *Metacyclops gasparoi* it is 0.6, *Metacyclops hannensis* and *Metacyclops subdolus* have a proportion ranging between 0.8 and 0.9. *Metacyclops agnitus* and *Metacyclops curtispinosus* shares similar values (1.1–1.2) and both *Metacyclops pectiniatus* and the new species have a length ratio close to 1.4 (Herbst 1988, Pesce 1985,Lim and Fernando 1985,Pesce 1978,Defaye 1992, Stoch 1987).

The length/width ratio of the caudal ramus also differs among these species, *Metacyclops curtispinosus* has relatively low value (2.4–2.8) that differs from those in *Metacyclops agnitus*, *Metacyclops pectiniatus*,and *Metacyclops hannensis* (3.2–3.4). *Metacyclops subdolus* has a wide range of variation (2.9–3.4). The new species has a relatively longer caudal ramus (3.5–3.8), but it is shorter than in *Metacyclops gasparoi* (5.5–5.7). Another valuable character is the length ratio of innermost terminal seta/outermost terminal seta; we found two main groups for this character. In the first one the innermost terminal seta is shorter than the outermost terminal seta; this character is present in *Metacyclops agnitus* (0.5), *Metacyclops pectiniatus* (0.6), *Metacyclops hannensis* (0.7),and inthe new species(0.8). In the second group the innermost terminal seta is longer than outermost terminal seta: *Metacyclops curtispinosus* (1.2), *Metacyclops subdolus* (1.5–1.7), and *Metacyclops gasparoi* (2.0). In addition, the length dorsal seta/length of caudal ramus ratio also separates two groups. In the first group, the dorsal seta is shorter than the ramus: *Metacyclops deserticus* sp. n. (0.4–0.5), *Metacyclops curtispinosus* and *Metacyclops hannensis* (0.7). In the second group the dorsal seta is longer than caudal ramus like in *Metacyclops gasparoi*, *Metacyclops agnitus* (1.1), and *Metacyclops subdolus* (1.8–2.1).

## Discussion

*Metacyclops deserticus* sp. n. from northern central Mexico represents the first new species of the genus *Metacyclops* described in this country and is also the third record for North America (Reid 1991, Reid and Williamson 2010). There is a previous record of *Metacyclops cushae* by Gutiérrez-Aguirre and Cervantes-Martínez (2013) from southeast Mexico (Chiapas State). Most of the known American species of *Metacyclops* occur in tropical environments of the Neotropical region, mainly in South America and only *Metacyclops cushae* and *Metacyclops gracilis* have been reported from the Neartic region (Dussart and Defaye 2006). All previous records of species of *Metacyclops* in the Americas are from tropical environments; the North American records are from marshes in Louisiana and the Everglades in Florida (Bruno et al. 2005, Reid 1991). The finding of this species from an arid environment in Mexico represents the first continental record of the genus from this kind of habitat (Dussart and Defaye 2006, Rocha 1994). Members of *Metacyclops* have been recorded from arid environments in other regions of the world like Australia, New Zealand and Africa; some of these species inhabit epigean systems but others are known from subterranean waters (Dussart 1977, Dumont 1981, Defaye 1992, Pesce et al. 1996, Karanovic 2004a, 2004b). According to Defaye (1992) species of *Metacyclops* usually inhabit small pools, wells and ponds rich in vegetation and organic material, and their presumed tolerance to high temperatures and salinities appear to predict a wider distribution in Africa and other places with arid conditions, now including the American desert systems.

The arid areas of north-central Mexico were formed between the Late Oligocene and Middle Miocene (30-20 MYA), and were part of a general trend toward a greater aridity resulting from climate changes associated to the intense volcanic activity and tectonics that characterized the Cenozoic. The Rocky Mountains, the Mexican and Central-American Plateaus, and the sierras Madre were formed as result of tectonic activity during Cenozoic. The formation of the Sierra Madre Occidental and Sierra Madre Oriental during the Eocene and continuing until the middle Miocene provided a new barrier to the atmospheric flow. This barrier blocked the masses of warm, moist air from the Pacific Ocean and the Gulf of Mexico and caused a severe drought and desertification of the Mexican Plateau. The Mexican Plateau includes the states of Coahuila, Chihuahua, Zacatecas, Durango. The Miocene climate change segregated the species along latitudinal and longitudinal gradients, thus favoring radiation processes of some lineages (Devitt 2006). It has been suggested that some of these areas of Northern Mexico functioned as refugia during the Pleistocene glaciations, thus favoring local process of isolation-speciation of the aquatic biota (Bănărescu 1991, Suárez-Morales et al. 2010, Mercado-Salas et al. 2012).

The cyclopoid copepod fauna of arid areas of central-north of Mexico (Chihuahuan Desert) is currently represented by 39 species belonging to 12 genera. This binational zone is currently deemed as an area with a high endemicity; up to 20% of these species are endemic to these arid areas (Mercado-Salas et al. 2006, 2009, Mercado-Salas and Suárez-Morales 2009, 2011, Suárez-Morales and Walsh 2009, Suárez-Morales et al. 2010). The new species of *Metacyclops* appears to be endemic to the Chihuahuan Desert, thus incrementing the importance of this and other arid systems as refuges of an undescribed diversity that certainly deserves further study. Most interestingly, the type locality harbors at least three endemic crustacean species, the copepod *Metacyclops deserticus* and the large branchiopods anostraceans (fairy shrimps) *Branchinecta oterosanvicentei* Obregón-Barboza, Maeda-Martínez, García-Velazco and Dumont, 2002, and *Streptocephalus guzmani* Maeda-Martínez, Belk, Obregón-Barboza and Dumont, 1995.

## Supplementary Material

XML Treatment for Metacyclops deserticus
